# Supplementary material for: An ESR1-Related Gene Signature Identifies Head and Neck Squamous Cell Carcinoma with Imputed Susceptibility to Endocrine Therapy
Source: Int J Mol Sci. 2024 Jan 19;25(2):1244. doi: 10.3390/ijms25021244 (PMC10816965; doi:10.3390/ijms25021244)
Supplement: Supplementary file 1 [file ijms-25-01244-s001.zip › Supplementary Figures S1 and S2.pdf]

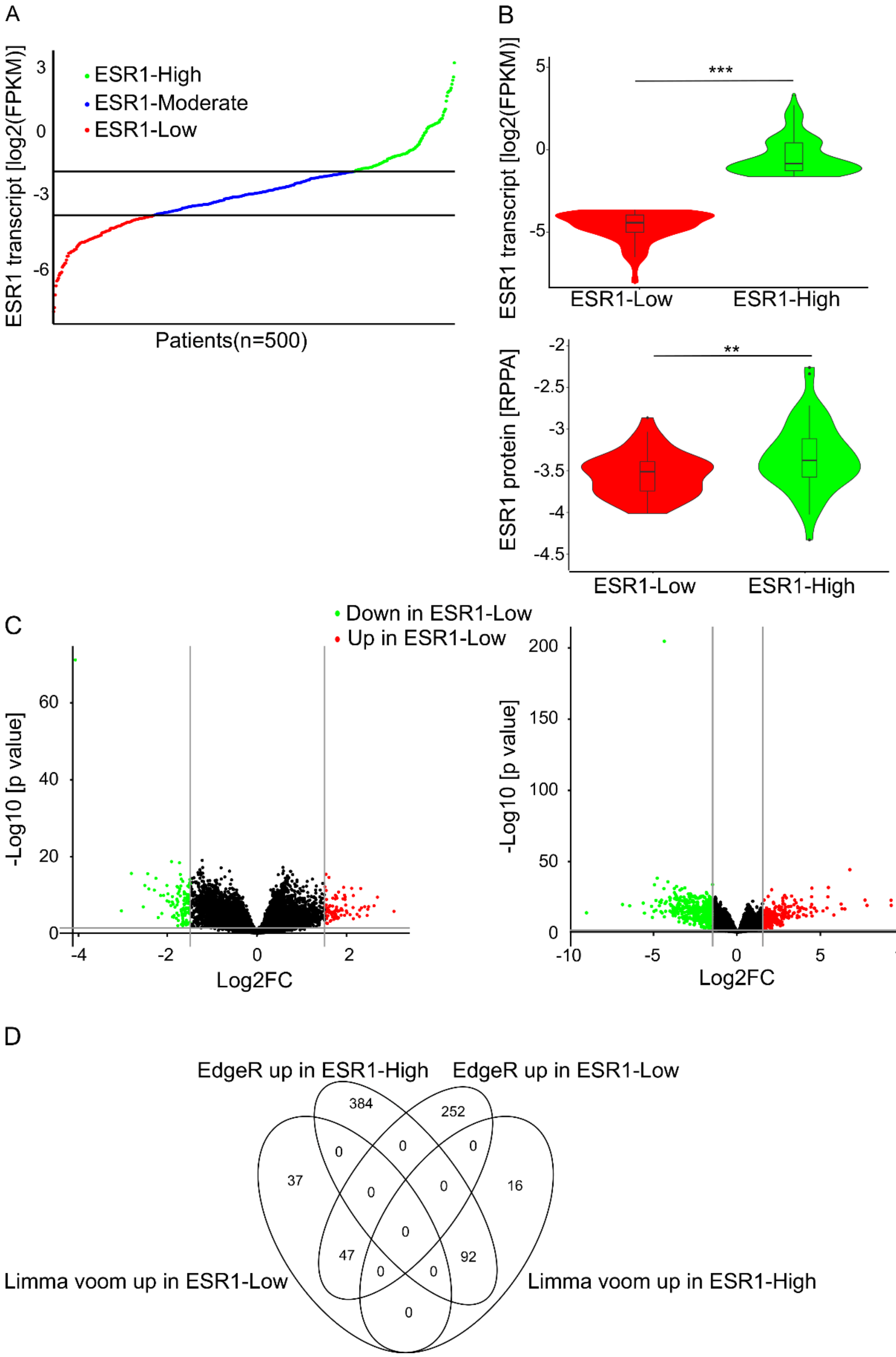

Supplementary Figure S1. ESR1-related DEGs in TCGA-HNSC. (**A**) Dot plot summarizes the distribution of ESR1 transcript values in the TCGA-HNSC cohort (n = 500) and selection of quartiles with higher (green) or lower ESR1 expression (red) for DEG analysis; (**B**) Violin plots confirm higher ESR1 transcript (top) and protein expression (bottom) in the ESR1-High compared to ESR1-Low quartile, respectively; (**C**) Volcano plots present significantly altered DEGs with either up- (red) or down-

regulation (green) among ESR1-High and ESR1-Low quartiles as determined by Limma-voom (left) or edgeR (right); (**D**) Venn diagram shows common DEGs for Limma-voom and EdgeR with either up- or down-regulation in the ESR1-High as compared to the ESR1-Low quartile. ** $p < 0.005$, *** $p < 0.0005$

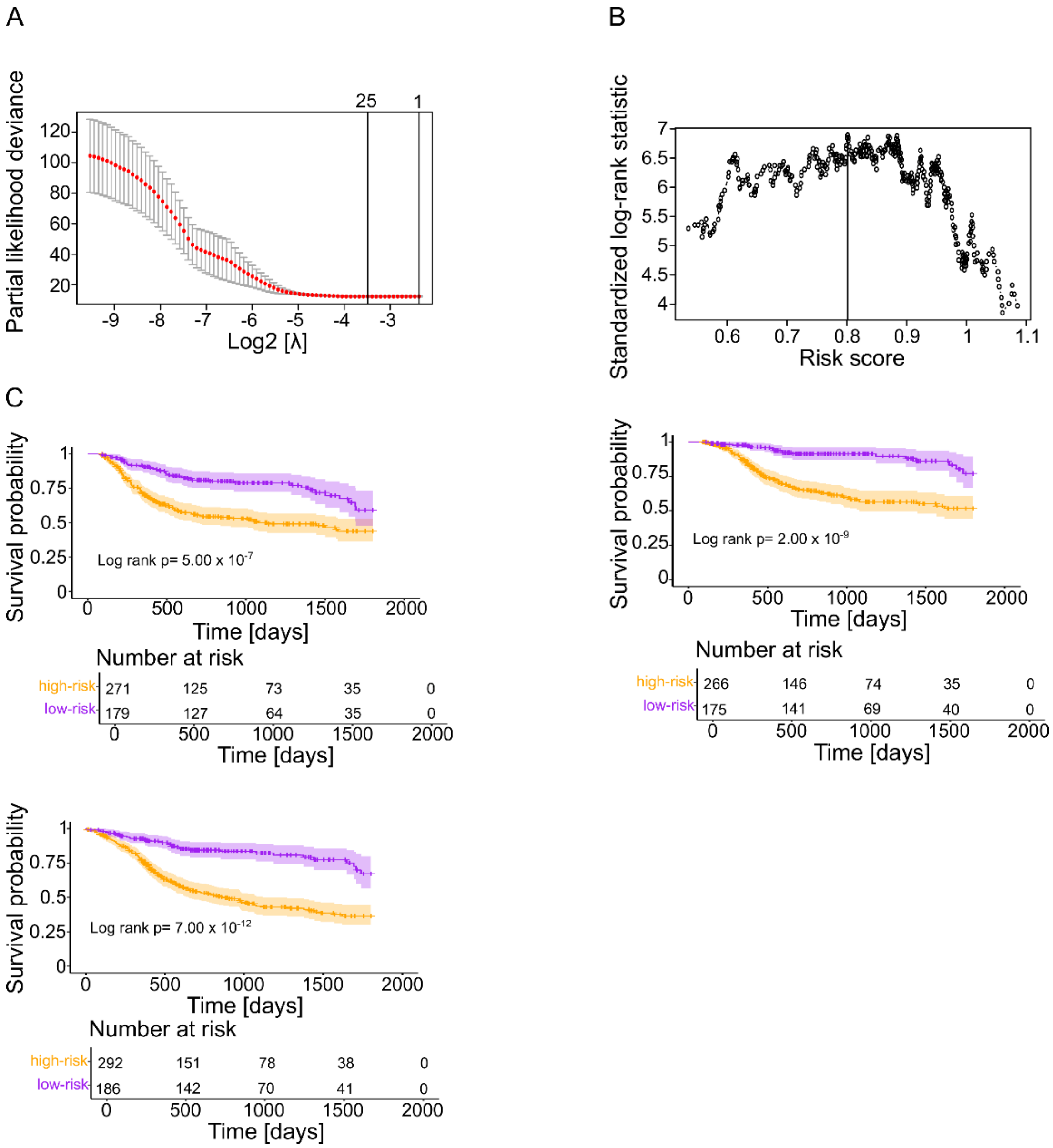

Supplementary Figure S2. The prognostic value of the HNSCC risk model. (**A**) The graph illustrates prioritization of most relevant DEGs concerning 5-years OS. The two vertical dotted lines represent the lambda.best and lambda.min cutoffs for selection of variables; (**B**) Coefficient values either negative (good prognosis) or positive (poor prognosis) were used to compute a risk score for individual cases. Patient stratification into high and low risk groups was done by best risk score cutoff; (**C**) Kaplan- Meier plots confirm unfavorable 5-years progression-free (upper left), disease-specific (upper right), and overall survival (lower) for the high-risk (orange line) as compared to the low-risk (purple line) group of TCGA-HNSC. Numbers of patients at risk at the indicated time points are given below each graph.
